# Social, Cognitive, and eHealth Mechanisms of COVID-19–Related Lockdown and Mandatory Quarantine That Potentially Affect the Mental Health of Pregnant Women in China: Cross-Sectional Survey Study

**DOI:** 10.2196/24495

**Published:** 2021-01-22

**Authors:** Xue Yang, Bo Song, Anise Wu, Phoenix K H Mo, Jiangli Di, Qian Wang, Joseph T F Lau, Linhong Wang

**Affiliations:** 1 The Jockey Club School of Public Health and Primary The Chinese University of Hong Kong Hong Kong China; 2 National Center for Women and Children's Health Chinese Center for Disease Control and Prevention Beijing China; 3 Department of Psychology University of Macau Macao Macao; 4 National Center for Chronic and Non-communicable Disease Control and Prevention Chinese Center for Disease Control and Prevention Beijing China

**Keywords:** eHealth, lockdown, quarantine, depression, anxiety, pregnant women

## Abstract

**Background:**

Although lockdown and mandatory quarantine measures have played crucial roles in the sharp decrease of the number of newly confirmed/suspected COVID-19 cases, concerns have been raised over the threat that these measures pose to mental health, especially the mental health of vulnerable groups, including pregnant women. Few empirical studies have assessed whether and how these control measures may affect mental health, and no study has investigated the prevalence and impacts of the use of eHealth resources among pregnant women during the COVID-19 outbreak.

**Objective:**

This study investigated (1) the effects of lockdown and mandatory quarantine on mental health problems (ie, anxiety and depressive symptoms), (2) the potential mediation effects of perceived social support and maladaptive cognition, and (3) the moderation effects of eHealth-related factors (ie, using social media to obtain health information and using prenatal care services during the COVID-19 pandemic) on pregnant women in China.

**Methods:**

An online cross-sectional survey was conducted among 19,515 pregnant women from all 34 Chinese provincial-level administrative regions from February 25 to March 10, 2020.

**Results:**

Of the 19,515 participants, 12,209 (62.6%) were subjected to lockdown in their areas of residence, 737 (3.8%) were subjected to mandatory quarantine, 8712 (44.6%) had probable mild to severe depression, 5696 (29.2%) had probable mild to severe anxiety, and 1442 (7.4%) had suicidal ideations. Only 640 (3.3%) participants reported that they used online prenatal care services during the outbreak. Significant sociodemographic/maternal factors of anxiety/depressive symptoms included age, education, occupation, the area of residence, gestational duration, the number of children born, complication during pregnancy, the means of using prenatal care services, and social media use for obtaining health information. Multiple indicators multiple causes modeling (*χ*^2^_14_=495.21; *P*<.05; comparative fit index=.99; nonnormed fit index=.98; root mean square error of approximation=.04, 90% CI 0.038-0.045) showed that quarantine was directly and indirectly strongly associated with poor mental health through decreased perceived social support and increased maladaptive cognition (B=.04; *β*=.02, 95% CI 0.01-0.02; *P*=.001), while lockdown was indirectly associated with mental health through increased social support and maladaptive cognition among pregnant women (B=.03; *β*=.03, 95% CI 0.02-0.03; *P*=.001). Multigroup analyses revealed that the use of social media for obtaining health information and the means of using prenatal care services were significant moderators of the model paths.

**Conclusions:**

Our findings provide epidemiological evidence for the importance of integrating mental health care and eHealth into the planning and implementation of control measure policies. The observed social and cognitive mechanisms and moderators in this study are modifiable, and they can inform the design of evidence-based mental health promotion among pregnant women.

## Introduction

### Background

Lockdown and mandatory quarantine are commonly used and effective measures that are implemented by governments to contain the transmission of respiratory infectious diseases, including the COVID-19 disease [1,2]. Lockdown refers to general and widespread restrictions on movement, work, and travel for all people in a city, region, or country. Lockdown measures include travel restrictions, the mandatory closure of schools, and bans on nonessential commercial and social activities. Given the global spread of COVID-19 to 216 countries/regions, which has resulted in over 30.6 million infections and 950,000 deaths as of September 20, 2020 [3], more than 100 countries (eg, the United States, France, Australia, Thailand, and South Africa) have adopted various forms of lockdown measures to control the pandemic [4]. In mainland China, more than 80 cities in around 20 provinces and municipalities were put in lockdown [5], and over 780 million people were under certain travel restrictions [6].

Mandatory quarantine is a form of isolating people who are not ill, but may have been exposed to a disease that is dangerous to society [7]. People who had close contact with individuals who were confirmed/suspected to have COVID-19, and people with a history of travel were quarantined for 14 days in designated facilities (eg, governmental facilities and hotels) or home settings [8]. Globally, more than 140 countries (eg, the United States, the United Kingdom, Spain, Italy, and Singapore) have adopted mandatory quarantine measures for disease control [9-12].

### The Impact of Lockdown and Mandatory Quarantine on Mental Health

Although lockdown and mandatory quarantine have played crucial roles in the sharp decrease of the number of newly confirmed/suspected COVID-19 cases [13-15], concerns have been raised over the threat that these measures pose to mental health, as these unprecedented measures have restricted daily routines and increased social isolation [16,17]. Empirical studies on the impact of lockdown/mandatory quarantine on mental health have yielded inconsistent results. According to the Office of National Statistics, more than 25 million people in the United Kingdom have experienced high levels of anxiety in late March 2020, which is when the lockdown was announced [17]. In a study conducted by Sibley et al [18], participants from New Zealand reported a slight increase in psychological distress, but less fatigue and no significant changes in rumination, feelings of belongingness, perceived social support, satisfaction with life, standards of living, future security, personal relationships, and health during the early phase of the nationwide lockdown compared to those during the pre-COVID-19 period. A study that was conducted in southern China during early February 2020 also found that people who were quarantined had a greater prevalence of anxiety and depression than those who were not affected by quarantine [19]. Furthermore, in mid-February 2020, Li et al [20] reported a positive association between perceived inconvenience to daily life caused by home quarantine and depression/anxiety among the general adult population in China. However, Zhu et al [21] reported that there was no significant differences in depression and anxiety between Chinese people who were and were not subjected to mandatory quarantine, and they concluded that although these mental health problems were not related to quarantine control measures, these measures did impact daily life. Another study in China even found a significantly lower prevalence of depression and anxiety among people under mandatory quarantine than among those who were infected by SARS-CoV-2 or the general public [22]. These inconsistent results highlight the importance of exploring potential underlying mechanisms (eg, the impacts of lockdown and quarantine on individuals) that may explain the relationship between lockdown and quarantine measures and mental health problems. We however did not identify such studies.

Pregnant women could be more susceptible to lockdown and quarantine measures and SARS-CoV-2 infection than the general population, due to their great need for social support and ongoing prenatal care services, concerns over fetal safety, immunocompromised status, and physiological and psychosocial adaptive changes during pregnancy [23]. Most studies on the mental health of pregnant women have small sample sizes (ie, 70 participants to around 560 participants), and these studies have only reported the prevalence of mental health problems during the COVID-19 pandemic [24-37]. We found 3 studies on the mental health of pregnant women that had larger sample sizes, with about ≥1000 respondents (ie, 946 participants to around 2421 participants) [24,25,30], and only 1 study (N=260) that investigated the impact of social isolation on mental health by simply asking pregnant women whether they believed that social isolation due to the pandemic affected their psychological well-being [27]. More studies that include large sample sizes and investigate the impact of lockdown/mandatory quarantine on the mental health of pregnant women during the COVID-19 pandemic are needed. Early detection and intervention can prevent the adverse impact that mental problems (eg, prenatal mental disorders) have on both mothers and children in the long term.

### Potential Social and Cognitive Mechanisms

Social and cognitive mechanisms may play critical roles in mediating the relationships between lockdown/mandatory quarantine and mental health. First, lockdown and quarantine are isolation measures that, by their nature, may induce social isolation and reduce social resources, such as social support, and reductions in such social resources are a risk factor of mental health problems [16]. We found 2 studies with a sample size that ranged between 308 participants to around 403 participants. These studies investigated the association between social support and mental health problems among pregnant women during the COVID-19 pandemic, and both studies reported negative associations [26,28]. The mediation effects of social support are supported by the conservation of resources theory, which predicts that resource loss (eg, losses of social resources like social support) is the principal factor in the stress process and the cause of mental disorder development [38].

Second, the governmental implementation of unprecedented measures for disease control may be a stressful event for the public that induces maladaptive cognitive responses. According to the response styles theory, both depression and anxiety are related to faulty cognitive responses to stressors and negative emotions [39,40]. Rumination (ie, the repetition of the same feelings and thoughts) and catastrophizing (ie, having thoughts that explicitly emphasize the terror of what one has experienced) are common maladaptive cognitions, and responses to maladaptive cognitions emerge when individuals experience threatening and uncertain events [41]. When compared to the different kinds of maladaptive cognitive responses, anxiety has been found to be more related to catastrophizing, which focuses on future threats [42], and depression has been found to be more related to ruminative thinking, which concentrates on past negative experiences and emotions [43,44]. Empirical studies have also supported the mediation roles of rumination and catastrophizing between threatening events (eg, daily hassles) and depression/anxiety [41,45]. No study has assessed the mediation effects of rumination and catastrophizing in the context of COVID-19 or pregnant women.

### eHealth-Related Moderators

eHealth refers to information and communications technologies in health care and the community. eHealth can be an optimal communication modality for people under stay-at-home orders, especially for those with time-sensitive health conditions, such as pregnancy [46]. eHealth and telemedicine services enable pregnant women to maintain their regular prenatal visit schedule and avoid the unnecessary risk of COVID-19 exposure [47]. Such online services may significantly affect individuals’ coping resources, stress appraisals, and perceived or actual social support [48,49], and may reduce the influence of external and environmental stress on individuals’ mental health during the COVID-19 pandemic. Thus, we hypothesized that the effects of control measures on interpersonal resources (eg, social support), cognitive status (eg, maladaptive cognition), and mental health might vary between pregnant women who use eHealth resources, such as using social media to obtain health information and online services to make appointments with doctors for prenatal care services during the COVID-19 pandemic, and those who did not use such resources. In addition, such extra resources may reduce the adverse effects of maladaptive cognition and enhance the protective effects of social support on mental health. We did not find any research that investigated the prevalence of eHealth resource use among pregnant women and the impact of using eHealth resources on the mental health of pregnant women.

### Objectives

This study aimed to investigate the prevalence of mental health problems (ie, depression and anxiety) among a large sample of pregnant women recruited from multiple regions in mainland China. We also assessed the direct and indirect effects of lockdown and mandatory quarantine on mental health problems through perceived social support and maladaptive cognition. We hypothesized that lockdown (ie, model path H1) and mandatory quarantine (ie, model path H2) would be positively associated with mental health problems. We further hypothesized that lockdown and mandatory quarantine would be indirectly associated with mental health problems through reduced perceived social support (ie, model path H3) and increased maladaptive cognition (ie, model path H4). Moreover, this study aimed to assess the moderation effects of eHealth-related variables, including using social media to obtain health information, using online prenatal care services, and making appointments with doctors during the COVID-19 outbreak, on pregnant women for each model path.

## Methods

### Participant Recruitment and Procedure

In this study, the inclusion criteria for the sample were (1) female sex, (2) age ≥18 years, (3) the ability to speak Chinese, (4) current pregnancy, and (5) the use of maternal health care services provided by the Maternal and Child Health Hospitals of the Chinese Preventive Medicine Association. Pregnant women who planned to terminate their pregnancy were excluded from this study. An online cross-sectional survey was conducted from February 24 to March 10, 2020. Eligible participants were identified from the records of Maternal and Child Health Hospitals from multiple regions of China and were invited to take part in the online survey by doctors through WeChat (Tencent Inc), which is the most widely used social media platform in Chinese populations. Interested participants visited the online survey through a link or quick response code and read the informed consent form before starting the survey. They were informed that clicking the “starting the survey” button implied informed consent. They were also informed that the study was anonymous and confidential, and that refusal to take part in the survey would not affect any services they would obtain. The survey took about 15 minutes to complete. No incentive was provided. A total of 19,515 pregnant women from all 34 provincial-level administrative regions in China (eg, 2127 pregnant women from Beijing, 4015 from Shandong, 3659 from Zhejiang, 1886 from Guangdong, 1250 from Hunan, 3178 from Shanxi, etc) completed the survey, with a valid response rate of 87.7%. This study was approved by the Survey and Behavioural Research Ethics Committee of the Chinese University of Hong Kong (Number SBRE-19-395).

### Measures

Sociodemographic and maternal information, including age, education level, occupation, the area of residence, gestational duration, the number of children born, complication during pregnancy, the major means of using prenatal care service during the COVID-19 pandemic (ie, using online services, making appointments with doctors, and going to a hospital as usual), and the frequency of using social media to obtain health information in the past week, were obtained from the survey. Participants also reported on their lockdown and mandatory quarantine status by answering the following questions: (1) “Had the city, town, or county where you currently live been put under lockdown by the local government because of the COVID-19 epidemic” (response score: 0=no and 1=yes); and (2) “Had you been under mandatory quarantine (e.g., governmental facilities-, hotel- or home-quarantine) because of the COVID-19 epidemic” (response score: 0=no and 1=yes)?

Perceived social support was measured by 2 dimensions (ie, general social support and perceived change in social support during the COVID-19 pandemic compared to those during the pre-COVID-19 period). The following 2 survey items were used to assess this: (1) “Overall, to what extent did you receive social support from families, friends, and others during the COVID-19 epidemic” (response scale: 1=very poor to 10=very good); and (2) “To what extent did your social support become poorer or better during COVID-19, compared to that before the outbreak of COVID-19” (response scale: 1=much poorer to 5=much better)? Similar survey items have been used in previous studies [50].

Maladaptive cognition related to COVID-19 was measured with the short-form Cognitive Emotion Regulation Questionnaire (CERQ) [51]. The CERQ was developed to evaluate the cognitive aspects of emotion regulation when one experiences stressful or unpleasant events. Sample items from the CERQ include “I am preoccupied with what I think and feel about what I have experienced” and “I keep thinking about how terrible it is what I have experienced.” These items are rated on Likert scales (ie, 1=almost never to 5=almost always). The subscales of rumination and catastrophizing were used in this study. The Chinese version has been validated in previous studies [52]. The reliability of the 2 subscales was acceptable for our sample (Cronbach α=.66; Cronbach α=.84, respectively).

Depression was measured using the Patient Health Questionnaire-9 (PHQ-9) [53]. Respondents evaluated the presence (PHQ-9 response scale: 0=none to 3=almost every day) of 9 criteria for a depressive episode that occurred in the past 2 weeks, in accordance with the Diagnostic and Statistical Manual of Mental Disorders, Fourth Edition (DSM-IV) (eg, “Feeling bad about yourself—or that you are a failure or have let yourself or your family down?”). The cutoff points for mild, moderate, moderately severe, and severe depression were total PHQ-9 scores of 5, 10, 15, and 20, respectively. The Chinese version has been used in previous studies [54], and it had good internal consistency (Cronbach α=.83).

Anxiety was measured by the Generalized Anxiety Disorder 7-item (GAD-7) scale [55]. It is based on DSM-IV criteria and is used to measure the severity of generalized anxiety disorder based on the past 2 weeks. Participants respond according to a 4-point Likert-type scale (ie, 0=none to 3=almost every day). The cutoff points for mild, moderate, and severe anxiety were total GAD-7 scores of 5, 10, and 15 respectively. The Chinese version has been validated in previous studies [56]. It had a Cronbach α of .90 with our sample.

### Data Analyses

Descriptive statistics, including frequency, means, and standard deviations, were computed for participants’ sociodemographic characteristics. Differences in depression scores and anxiety scores based on sociodemographic characteristics were compared by either an independent 2-tailed *t* test or analysis of variance. The effect size (ie, Cohen *d* or Cohen *f*) was reported. Bivariate correlations between the key variables were presented. The effect size was considered low if the value of *r* varied around .10, medium if *r* varied around .30, and large if *r* varied by more than .50 [57]. Multiple indicators multiple causes (MIMIC) modeling was conducted to test the proposed mediation model. The goodness of fit was tested, and standardized path coefficients (ie, *β*) were reported. The mediation hypotheses were tested by bootstrapping analyses. The 95% confidence intervals of the indirect effects were obtained from 5000 bootstrap samples. The effect size (ie, proportion of mediation [PM]) was reported. Multigroup analyses were conducted to test the proposed moderators; *P*<.05 in the Chi-square difference test (Δχ^2^/Δdf) would suggest a significant moderation effect. The missing data rate was below 5%, and all missing values were replaced by using multiple imputation. The level of statistical significance was .05, and SPSS version 21.0 (IBM Corp) and Amos Version 26 (IBM Corp) were used for data analyses.

## Results

### Sociodemographic and Maternal Characteristics

Tables 1-3 present the sociodemographic and maternal characteristics of the participants. Of 19,515 participants, 13,885 (71.1%) were aged 26-35 years, 11,627 (59.6%) had an education level of college or above, 10,741 (55%) did not have a baby before this pregnancy, and 17,856 (91.5%) did not experience any complications or comorbidities during pregnancy. Additionally, 5394 (27.6%) participants were unemployed/housewives (27.6%) and 5193 (26.6) were technical/administrative personnel (26.6%). The distribution of participants’ areas of residence was approximately even (municipality/provincial capitals: n=5930, 30.4%; general cities: n=6855, 35.1%; counties: n=6730, 34.5%). Participants’ average gestational duration was 25.4 weeks (SD 9.8 weeks).

**Table 1:** Sociodemographic and maternal characteristics of the participants (N=19,515).

Characteristics		Value
**Age (years), n (%)**		
	<26	3781 (19.4)
	26-30	8202 (42)
	31-35	5683 (29.1)
	>35	1849 (9.5)
**Education, n (%)**		
	Middle school or below	4014 (20.6)
	High school	3874 (19.9)
	College	5222 (26.8)
	University or above	6405 (32.8)
**Occupation, n (%)**		
	Technical personnel	3053 (15.6)
	Administrative personnel	2140 (11)
	Civil servant	320 (1.6)
	Soldier	22 (0.1)
	Business/service personnel	1778 (9.1)
	Self-employed/private business owner	1455 (7.5)
	Farmer/migrant worker	746 (3.8)
	Unemployed/housewife	5394 (27.6)
	Student (undergraduate/postgraduate)	51 (0.3)
	Other	4556 (23.3)
**Area of residence, n (%)**		
	Municipality/provincial capital	5930 (30.4)
	General city	6855 (35.1)
	County	6730 (34.5)
Number of gestational weeks, mean (SD)		25.4 (9.8)
**Gestational duration (weeks), n (%)**		
	1-10	1523 (7.8)
	11-20	4986 (25.5)
	21-30	5858 (30)
	>30	6518 (33.4)
	Not sure	630 (3.2)
**Number of children born before pregnancy, n (%)**		
	0	10741 (55)
	1	7796 (39.9)
	>1	978 (5)
**Complication during pregnancy, n (%)**		
	Yes	1659 (8.5)
	No	17856 (91.5)

**Table 2:** Sociodemographic and maternal characteristics of the participants (N=19,515) based on eHealth-related variables.

eHealth-related variables		Value
**Means of using prenatal care service, n (%)**		
	Went to hospital as usual	10189 (52.2)
	Made appointment with doctor	7568 (38.8)
	Online prenatal care	640 (3.3)
	Not sure	1118 (5.7)
**Used social media to obtain health information, n (%)**		
	Never	1781 (9.1)
	Sometimes	10605 (54.3)
	Always	7129 (36.6)

With regard to the means of using prenatal care services during the COVID-19 epidemic, 10,189 (52.2%) participants went to the hospital for prenatal care as usual, 7568 (38.8%) made appointments with doctors, 640 (3.3%) used online services, and 1118 (5.7%) were uncertain. Most participants (n=17,734, 90.9%) used social media to obtain health information in the week before the survey.

Of the 19,515 participants, 12,209 (62.6%) participants reported lockdown in their areas of residence; 737 (3.8%) were subjected to mandatory quarantine; 8712 (44.6%) had probable mild to severe depression; 1442 (7.4%) had suicidal ideations, as measured by question 9 in the PHQ-9; and 5696 (29.2%) had probable mild to severe anxiety.

**Table 3:** Sociodemographic and maternal characteristics of the participants (N=19,515) based on psychosocial variables.

Psychosocial variables		Value
**Lockdown in the area of residence, n (%)**		
	Yes	12209 (62.6)
	No	7306 (37.4)
**Subjected to quarantine, n (%)**		
	Yes	737 (3.8)
	No	18778 (96.2)
General social support, mean (SD)		8.51 (2.07)
Social support change, mean (SD)		4.05 (1.06)
Rumination, mean (SD)		3.11 (0.86)
Catastrophizing, mean (SD)		2.76 (1.09)
Depressive symptoms, mean (SD)		0.56 (0.56)
**Depressive symptoms (total PHQ-9^a^ score), n (%)**		
	Minimal (0-4)	10803 (55.4)
	Mild (5-9)	5565 (28.5)
	Moderate (10-14)	2053 (10.5)
	Moderately severe (15-19)	793 (4.1)
	Severe (20-27)	301 (1.5)
Anxiety symptoms, mean (SD)		0.46 (0.60)
**Anxiety symptoms (total GAD-7^b^ score), n (%)**		
	Minimal (0-4)	13819 (70.8)
	Mild (5-9)	4177 (21.4)
	Moderate (10-14)	1052 (5.4)
	Severe (15-21)	467 (2.4)
Self-harm/suicidal ideation^c^, mean (SD)		0.10 (0.41)
**Self-harm/suicidal ideation frequency^c^, n (%)**		
	None	18073 (92.6)
	Several days	979 (5.0)
	More than half of the days	325 (1.7)
	Almost every day	138 (0.7)

^a^PHQ-9: Patient Health Questionnaire-9.

^b^GAD-7: Generalized Anxiety Disorder 7-item.

^c^Based on item 9 in the Patient Health Questionnaire-9.

### Level of Mental Health Problems Based on Sociodemographic Characteristics

As seen in Tables 4 and 5, factors that positively significantly associated with both depressive and anxiety symptoms included young age, being a student, residing in counties, being in the early or final stages of pregnancy, using means of prenatal care other than making appointments with doctors, using social media to obtain health information, experiencing lockdown in the areas of residence, and being quarantined. Other factors that were significantly associated with greater anxiety included low education levels (*P*=.01) and complications during pregnancy (*P*<.001). With regard to the number of children born, participants who were giving birth for the first time reported significantly more depressive symptoms than those who were not giving birth for the first time (*P*<.001). They also reported more anxiety symptoms than those who had given birth to 1 child before this pregnancy (*P*=.002).

**Table 4:** Depressive and anxiety symptoms stratified by sociodemographic and maternal characteristics.

Variables		Depressive symptoms				Anxiety symptoms			
		Mean (SD)	*F* test (df) or *t* test (df)	*P* value	Cohen *d* or Cohen *f*	Mean (SD)	*F* test (df) or *t* test (df)	*P* value	Cohen *d* or Cohen *f*
**Age (years)**		N/A^a^	34.95 (319,511)	<.001	0.07	N/A	18.12 (319,511)	<.001	0.05
	<26	0.62 (0.62)				0.51 (0.67)			
	26-30	0.56 (0.54)				0.45 (0.58)			
	31-35	0.54 (0.54)				0.45 (0.59)			
	>35	0.57 (0.52)				0.40 (0.57)			
**Education**		N/A	2.30 (319,511)	.08	0.02	N/A	4.17 (319,511)	.01	0.03
	Middle school or below	0.55 (0.61)				0.48 (0.66)			
	High school	0.58 (0.60)				0.47 (0.63)			
	College	0.56 (0.54)				0.44 (0.57)			
	University or above	0.55 (0.51)				0.45 (0.57)			
**Occupation**		N/A	3.80 (919,505)	<.001	0.04	N/A	3.35 (919,505)	<.001	0.04
	Technical staff	0.55 (0.51)				0.44 (0.57)			
	Administrative staff	0.54 (0.51)				0.44 (0.57)			
	Civil servant	0.62 (0.56)				0.52 (0.66)			
	Soldier	0.45 (0.45)				0.32 (0.40)			
	Business/service personnel	0.57 (0.55)				0.45 (0.58)			
	Self-employed/private business owner	0.56 (0.57)				0.45 (0.59)			
	Farmer/migrant worker	0.51 (0.60)				0.43 (0.63)			
	Unemployed/housewife	0.58 (0.59)				0.48 (0.63)			
	Student (undergraduate/postgraduate)	0.75 (0.66)				0.69 (0.71)			
	Other	0.54 (0.56)				0.45 (0.60)			
**Area of residence**		N/A	12.05 (219,512)	<.001	0.04	N/A	8.24 (219,512)	<.001	0.03
	Municipality/provincial capital	0.53 (0.52)				0.44 (0.47)			
	General city	0.44 (0.56)				0.45 (0.60)			
	County	0.58 (0.59)				0.48 (0.63)			
**Gestational duration (week)**		N/A	10.64 (419,510)	<.001	0.04	N/A	9.71 (419,510)	<.001	0.04
	1-10	0.62 (0.61)				0.48 (0.64)			
	11-20	0.57 (0.55)				0.44 (0.59)			
	21-30	0.53 (0.54)				0.43 (0.58)			
	>30	0.56 (0.55)				0.48 (0.61)			
	Not sure	0.56 (0.67)				0.54 (0.73)			
**Number of children born before pregnancy**		N/A	17.01 (219,512)	<.001	0.04	N/A	6.34 (219,512)	.002	0.02
	0	0.58 (0.54)				0.47 (0.60)			
	1	0.53 (0.57)				0.44 (0.60)			
	>1	0.52 (0.63)				0.48 (0.80)			
**Complication during pregnancy**		N/A	0.03 (19,513)	0.98	0.00	N/A	3.70 (1961)	<.001	0.10
	Yes	0.56 (0.55)				0.51 (0.62)			
	No	0.56 (0.56)				0.45 (0.60)			

^a^N/A: not applicable.

**Table 5:** Depressive and anxiety symptoms stratified by eHealth-related variables.

Variables		Depressive symptoms				Anxiety symptoms			
		Mean (SD)	*F* test (df) or *t* test (df)	*P* value	Cohen *d* or Cohen *f*	Mean (SD)	*F* test (df) or *t* test (df)	*P* value	Cohen *d* or Cohen *f*
**Means of using prenatal care services**		N/A^a^	29.35 (319,511)	<.001	0.06	N/A	23.66 (319,511)	<.001	0.06
	Went to hospital as usual	0.57 (0.56)				0.46 (0.61)			
	Made appointment with doctor	0.52 (0.53)				0.42 (0.57)			
	Online prenatal care	0.60 (0.62)				0.50 (0.65)			
	Not sure	0.67 (0.63)				0.58 (0.69)			
**Used social media to obtain health information**		N/A	3.69 (219,512)	.03	0.03	N/A	3.06 (219,512)	.04	0.03
	Never	0.52 (0.61)				0.43 (0.66)			
	Sometimes	0.56 (0.55)				0.45 (0.59)			
	Always	0.56 (0.55)				0.47 (0.60)			

^a^N/A: not applicable.

### Bivariate Correlations Between the Key Psychosocial Variables

As seen in Table 6, living in an area under lockdown had significant positive associations with all the key psychological variables, including perceived general social support (*P*<.001), social support change (*P*<.001), rumination (*P*<.001), catastrophizing (*P*<.001), depression (*P*=.01), and anxiety (*P*=.03). Being quarantined was significantly and negatively associated with perceived general social support (*P*<.001) and social support change (*P*=.01), while it was significantly and positively associated with catastrophizing (*P*<.001), depression (*P*<.001), and anxiety (*P*<.001). However, all the correlations associated with lockdown and quarantine had small effect sizes. Both perceived general social support and social support change had small to moderate negative correlations with depression (*r*=−0.17, *P*<.001; *r*=−0.16, *P*<.001, respectively) and anxiety (*r*=−0.17, *P*<.001; *r*=−0.15, *P*<.001, respectively). Both rumination and catastrophizing had moderate positive correlations with depression (*r*=0.25, *P*<.001; *r*=0.28, *P*<.001, respectively) and anxiety (*r*=0.26, *P*<.001; *r*=0.31, *P*<.001, respectively).

**Table 6:** Bivariate correlations (Spearman ρ, Pearson *r*, and *P* values) between the key psychosocial variables.

Variable		Lockdown	Quarantine	General social support	Social support change	Rumination	Catastrophizing	Depressive symptoms	Anxiety symptoms
**Lockdown**									
	ρ	1	0.06^a^	0.04^a^	0.06^a^	0.05^a^	0.09^a^	0.02^b^	0.02^c^
	*P* value	—^d^	<.001	<.001	<.001	<.001	<.001	.01	.03
**Quarantine**									
	ρ	0.06^a^	1	−0.03^a^	−0.02^b^	0.01	0.03^a^	0.04^a^	0.04^a^
	*P* value	<.001	—	<.001	.01	.12	<.001	<.001	<.001
**General social support**									
	*r*	—	—	1	0.64^a^	0.05^a^	−0.04^a^	−0.17^a^	−0.17^a^
	*P* value	—	—	—	<.001	<.001	<.001	<.001	<.001
**Social support change**									
	*r*	—	—	0.64^a^	1	0.09^a^	0.02^c^	−0.16^a^	−0.15^a^
	*P* value	—	—	<.001	—	<.001	.02	<.001	<.001
**Rumination**									
	*r*	—	—	0.05^a^	0.09^a^	1	0.66^a^	0.25^a^	0.26^a^
	*P* value	—	—	<.001	<.001	—	<.001	<.001	<.001
**Catastrophizing**									
	*r*	—	—	−0.04^a^	0.02^c^	0.66^a^	1	0.28^a^	0.31^a^
	*P* value	—	—	<.001	.02	<.001	—	<.001	<.001
**Depressive symptoms**									
	*r*	—	—	−0.17^a^	−0.16^a^	0.25^a^	0.28^a^	1	0.78^a^
	*P* value	—	—	<.001	<.001	<.001	<.001	—	<.001
**Anxiety symptoms**									
	*r*	—	—	−0.17^a^	−0.15^a^	0.26^a^	0.31^a^	0.78^a^	1
	*P* value	—	—	<.001	<.001	<.001	<.001	<.001	—

^a^The correlation is significant at a level of .001.

^b^The correlation is significant at a level of .01.

^c^The correlation is significant at a level of .05.

^d^Not applicable.

### MIMIC Modeling Analyses for the Proposed Model

**Figure 1:**
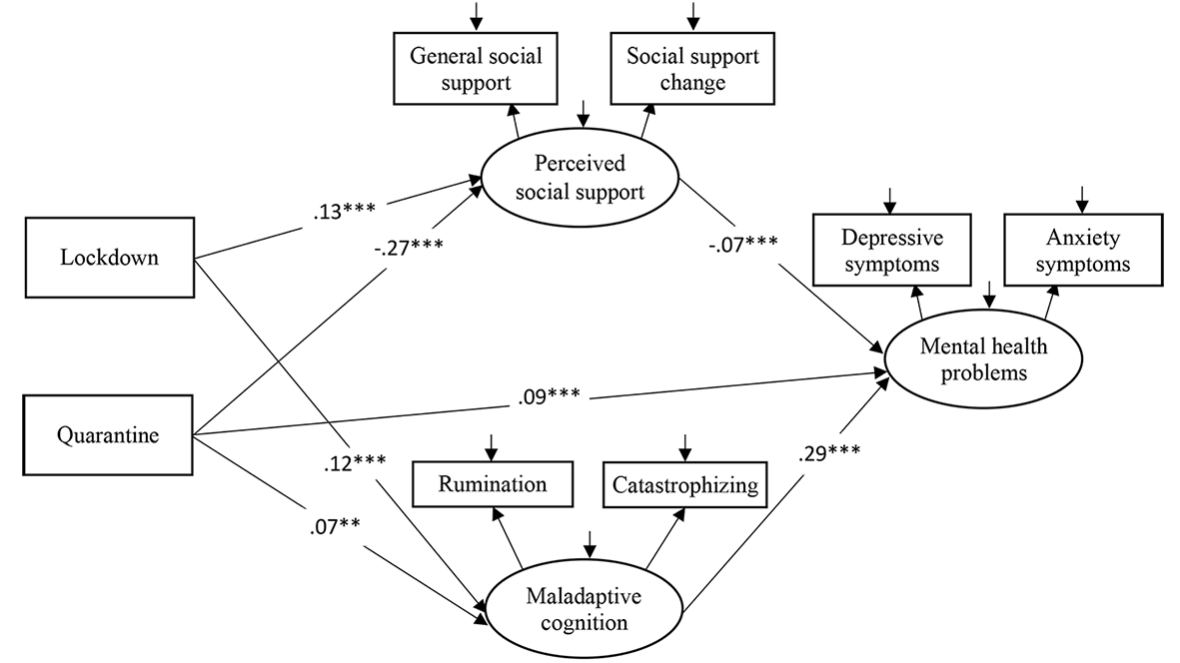
Proposed mediation model with unstandardized path coefficients. The nonsignificant path is not shown for simplicity reasons. *P<.05, **P<.01, ***P<.001.

MIMIC modeling showed that both the measurement model (χ^2^_12_=411.75; comparative fit index [CFI]=.99; nonnormed fit index [NNFI]=.98; root mean square of error approximation [RMSEA]=.04, 90% CI 0.038-0.045) and structural model (χ^2^_14_=495.21; CFI=.99; NNFI=.98; RMSEA=.04, 90% CI 0.038-0.045) (Figure 1) fit the data well. Lockdown was significantly and positively associated with perceived social support (B=.13; *β*=.04; *P*<.001) and maladaptive cognition (B=.12; *β*=.09; *P*<.001), but not with mental health problems (B=.01; *β*=.01; *P*=.33). Mandatory quarantine was associated with low perceived social support (B=−.27; *β*=−.03; *P*<.001), great maladaptive cognition (B=.07, *β*=.02, *P*=.01), and great mental health problems (B=.09; *β*=.04; *P*<.001). Perceived social support was negatively associated with mental health problems (B=−.07; *β*=−.23; *P*<.001), while maladaptive cognition was positively associated with mental health problems (B=.29; *β*=.39; *P*<.001). Detailed results regarding regression weights are provided in Multimedia Appendix 1.

### Mediation Test

The indirect effects of lockdown (B=.03; *β*=.03, 95% CI 0.02-0.03; *P*=.001; PM=78.8%) and quarantine (B=.04; *β*=.02, 95% CI 0.01-0.02; *P*=.001; PM=30.1%) on mental health problems were statistically significant. Specifically, the indirect effect of lockdown on mental health problems through perceived social support was negative (B=−.01; *β*=−.01; *P*<.001), and the indirect effect of lockdown on mental health problems through maladaptive cognition was positive (B=.03, *β*=.04, *P*<.001). The indirect effects of quarantine on mental health problems through perceived social support (B=.02; *β*=.01; *P*<.001) and maladaptive cognition (B=.02; *β*=.01; *P*<.001) were positive. The total effects of lockdown (B=.03; *β*=.03; *P*=.001) and quarantine (B=.13; *β*=.05; *P*=.001) on mental health problems were statistically significant.

### Moderation Test

The use of social media for obtaining health information during the COVID-19 pandemic significantly moderated the association between perceived social support and mental health problems (Δ*χ*^2^=18.58, Δdf=2) (Multimedia Appendix 2). Specifically, the negative associations between perceived social support and mental health problems became stronger with the increased frequency of using social media (never: B=−.05; *β*=−.18; *P*<.001; sometimes: B=−.06; *β*=−.22; *P*<.001; always: B=−.08; *β*=−.27; *P*<.001).

The means of using prenatal care services during the COVID-19 pandemic significantly moderated the 3 model paths (Multimedia Appendix 3), including the paths from lockdown to maladaptive cognition (Δ*χ*^2^=20.10, Δdf=3; went to hospital as usual: B=.16; *β*=.12; *P*<.001; made appointment with doctor: B=.07; *β*=.05; *P*<.001; online prenatal care: B=.13; *β*=.09; *P*=.03; uncertain: B=.05; *β*=.04; *P*=.25), the paths from mandatory quarantine to perceived social support (Δ*χ*^2^=8.12, Δdf=3; went to hospital as usual: B=−.30; *β*=−.03; *P*=.004; made appointment with doctor: B=−.07; *β*=−.10; *P*=.504; online prenatal care: B=−.78; *β*=−.12; *P*=.01; uncertain: B=−.43; *β*=−.06; *P*=.07), and the paths from maladaptive cognition to mental health problems (Δ*χ*^2^=11.48, Δdf=3; went to hospital as usual: B=.30; *β*=.41; *P*<.001; made appointment with doctor: B=.26; *β*=.37; *P*<.001; online prenatal care: B=.35; β=.42; *P*<.001; uncertain: B=.32; *β*=.38; *P*<.001).

## Discussion

In this large-scale study, we report on the high prevalence of depression and anxiety in Chinese pregnant women during the COVID-19 pandemic and the potential sociodemographic, maternal, eHealth-related, control measure-related, cognitive, and social factors that affect them. Furthermore, we found that control measures were associated with depression and anxiety through social support and maladaptive cognition, and that the use of social media to obtain health information and the means of using prenatal care services were potential moderators of these associations.

Anxiety and depression are the most common mental disorders that occur during pregnancy, affecting between 10%-30% of pregnant women in China and other countries [58-61]. Our results suggest that the COVID-19 pandemic has resulted in a substantial increase in pregnant women’s risk of mental health problems, as 44.6% (8712/19,515) and 29.2% (5696/19,515) of our participants had probable mild to severe depression or anxiety, respectively. The significant background factors of anxiety/depression, including age, socioeconomic status, pregnancy-related status, and health service use, are in line with recent COVID-19 studies and non-COVID-19 studies [25,58,59,62]. In general, our results suggest that women with less social capital, experience in pregnancy, or health service resources/access experience more mental distress during the COVID-19 pandemic than those without such issues. In addition, this is the first study to reveal the associations between eHealth-related activities (ie, using online prenatal care services, making appointments with doctors, or using social media to obtain health information) and anxiety/depressive symptoms among pregnant women during the COVID-19 pandemic. We found that about half of the participants (10,189/19,515, 52.2%) went to hospital for prenatal care services as usual, while only 3.3% (640/19,515) used online prenatal care services. This may suggest that there is an urgent need to improve the quality of online prenatal care services and eHealth literacy and popularize the use of such services among pregnant women. These services allow pregnant women to access maternal health care with minimum COVID-19 exposure risk, which is desirable during the outbreak [63]. We found that 7.4% (1442/19,515) of pregnant women had self-harm/suicidal ideations in the past 2 weeks, which is slightly higher than the 5.2% prevalence rate reported before the COVID-19 epidemic [64]. Future studies should identify the causes of these mental health problems and the long-term impact of mental health problems on pregnant women. Tailored and timely interventions for mental health promotion are warranted, especially for vulnerable subgroups, such as pregnant women.

It is intriguing that in our study, lockdown and mandatory quarantine affected mental health problems in different ways through different underlying mechanisms. First, the total effect and indirect effects of lockdown on mental health problems were statistically significant, but the direct effects of lockdown were not. Lockdown increased the incidence of mental health problems through enhancing maladaptive cognition, which is consistent with previous studies on maladaptive cognition in the context of other stressful events (eg, daily hassles) [41,45]. Interestingly, we found that lockdown might increase perceived social support, which in turn might reduce the prevalence of mental health problems. However, a study in New Zealand found a nonsignificant change in perceived social support during the nationwide lockdown compared to that during the pre-COVID period [18]. The different associations between lockdown and perceived social support in the New Zealand study and our study may be partially due to the differences in study designs and samples, or the fact that the severity of lockdown measures and concomitant supporting measures varied across countries [1,2]. The positive association between lockdown and perceived social support in our study may be due to the fact that lockdown prohibits people from leaving an area, which might have increased participants’ time and opportunities to stay and communicate with their families. A study conducted on April 2020 in Ireland (N=70) also reported that pregnant women improved their relationships with their partners by talking more, exercising together, and sharing tasks during lockdown [29]. In addition, governmental support has increased during the COVID-19 pandemic, and this has had a protective effect against anxiety among Chinese residents [65]. Thus, increased family support and governmental support during lockdown might explain why lockdown had a positive association with perceived social support and a negative indirect effect on mental health problems among Chinese pregnant women. These explanations should be assessed in future work, such as qualitative studies and case studies.

Second, as hypothesized, mandatory quarantine was significantly associated with a greater incidence of mental health problems. This result is consistent with several previous studies [19,20,66,67]. Furthermore, we found that reduced perceived social support and increased maladaptive cognition may explain this association, which is consistent with a study conducted by Zhu et al [21], who argued that the impacts of quarantine on daily life may explain the effect of quarantine on mental health problems. Quarantine is different from regional or nationwide lockdowns, as quarantine means that individuals are not allowed to leave the building or receive visitors. This difference may partially explain why lockdown increased perceived social support and mandatory quarantine reduced perceived social support in our study. Mandatory quarantine may also substantially affect other aspects of daily life, such as difficulties with quarantine compliance, inadequate information on prevention measures, decreased physical activity, the perceived high risk of COVID-19 infection [68], increased concerns over fetal safety, and difficulties in receiving prenatal care [23], which in turn aggravate mental health problems among pregnant women. Future studies should investigate these potential mediators and identify the cause of postquarantine changes in mental health status (eg, posttraumatic stress disorder development and increased stress).

Our findings highlight the importance of social and cognitive mechanisms in understanding the associations between lockdown/mandatory quarantine and mental health. In terms of our sample, the mediation effects of social and cognitive mechanisms accounted for large proportions of the total effects in the model. In general, the mediation model is supported by the conservation of resources theory [38] and the response styles theory [39,40], which explain how control measures for COVID-19 influence mental health. Based on these theories, future studies may explore other mediators, such as the loss and gain of other types of resources (eg, financial/personal resources) and other types of cognitive responses (eg, positive reappraisal). It is particularly important to identify modifiable psychosocial mediators because COVID-19 might become a persistent health threat, and such control measures might be inevitable [69]. Furthermore, our findings have important practical clinical and political implications. Since referrals are not feasible in the context of the COVID-19 pandemic, mental health first aid and brief non-face-to-face intervention services, such as the screening of mental distress in high-risk groups, counselling hotlines, and online education for problem-solving and stress-coping skills, should be made available for pregnant women and other vulnerable populations. In addition, it is important to guarantee that pregnant women who stay in areas under lockdown or mandatory quarantine can maintain regular communication with their significant others through social media, and receive adequate social support from both significant others and health/social care staff. More intensive therapies, such as cognitive behavioral therapy for cognitive restructuring and adaptive skill training, may be needed for those who experience great anxiety or depressive symptoms, such as the 29.2% (5696/19,515) and 44.6% (8712/19,515) of participants who had probable mild to severe anxiety or depression in our sample, respectively. Several environmental and structural factors, such as adequate preventive facilities, timely and accurate health information, access to multiple health service resources, and mass publicity for properly promoting accurate information on COVID-19, may also help pregnant women to reduce maladaptive cognition and facilitate positive reappraisal. Our results highlight the importance of integrating mental health care and eHealth into the implementation of control measures.

We also found that the use of social media for obtaining health information and the means of using prenatal care services during the COVID-19 pandemic were significant moderators in the model paths. Specifically, social media use strengthened the protective effect of perceived social support on mental health, and making appointments with doctors for prenatal care might buffer the adverse effects of lockdown and mandatory quarantine on maladaptive cognition and perceived social support, respectively. Furthermore, making such appointments might also buffer the adverse effects of maladaptive cognition on mental health. Using social media and making appointments with doctors may imply that people have various ways to access multiple health information and health service resources, which play an important role in buffering the stress appraisal and coping processes. These alternatives may be particularly useful for pregnant women during the COVID-19 pandemic, as high-quality and ongoing prenatal care is essential in supporting a healthy pregnancy and detecting risks early [70]. They are also helpful in reducing the risk of infection and related concerns among pregnant women [46]. In general, our results suggest that virtual visits and telemedicine should be included as part of a bundled care model.

Ours is one of the very few studies to assess pregnant women who experience and do not experience lockdown/mandatory quarantine and explore how such experiences might influence pregnant women’s interpersonal, cognitive, and mental statuses. Although our study adds to the literature on disease control and mental research, it has several limitations. First, the cross-sectional design prohibits causal inferences; the mediation model is exploratory and should not be interpreted as a causal mediation model. Our findings are intended for the generation of future research questions and provision of preliminary insights for when longitudinal studies are less feasible. Second, given the large sample size, the associations between small effect sizes could be statistically significant. Therefore, the interpretation of our results should be based on both statistical significance and effect size. Third, single items were used to assess perceived general social support and social support change; psychometric properties could not be established. Finally, the nonrandomly selected sample may have introduced selection bias, and the generalization of the study findings to other populations should be made cautiously. However, the large sample was recruited from all 34 provincial-level administrative regions in China. Therefore, our sample may accurately represent the population of China at a national level.

In conclusion, quarantine was strongly directly and indirectly associated with poor mental health status through decreased perceived social support and increased maladaptive cognition, while lockdown was indirectly associated with mental health through increased perceived social support and maladaptive cognition among pregnant women. The use of social media for obtaining health information and the means of using prenatal care service were significant moderators in the model paths. Follow-up studies are warranted to examine the long-term impacts of lockdown/quarantine control measures. The Chinese context of this study and the present global situation may differ in terms of the number of confirmed cases, the types and severity of control measures, and public responses toward COVID-19 and control measures. The validation of our findings and identification of similarities and differences across different countries are warranted.

## Appendix

Multimedia Appendix 1 Model path results in Amos.

Multimedia Appendix 2 Using social media for obtaining health information as a moderator of the model paths.

Multimedia Appendix 3 Means of using prenatal care services as a moderator of the model paths.

## Abbreviations

CERQ: Cognitive Emotion Regulation Questionnaire
CFI: comparative fit index
DSM-IV: Diagnostic and Statistical Manual of Mental Disorders, Fourth Edition
GAD-7: Generalized Anxiety Disorder 7-item
MIMIC: multiple indicators multiple causes
NNFI: nonnormed fit index
PHQ-9: Patient Health Questionnaire-9
PM: proportion of mediation
RMSEA: root mean square of error approximation
