# Supplementary material for: Social, Cognitive, and eHealth Mechanisms of COVID-19–Related Lockdown and Mandatory Quarantine That Potentially Affect the Mental Health of Pregnant Women in China: Cross-Sectional Survey Study
Source: J Med Internet Res. 2021 Jan 22;23(1):e24495. doi: 10.2196/24495 (PMC7836909; doi:10.2196/24495)
Supplement: Multimedia Appendix 1 [file jmir_v23i1e24495_app1.docx]

Appendix 1: Model Path Results in Amos.

**Regression Weights: (Group number 1 - Default model)**

|  |  |  | **Estimate** | **S.E.** | **C.R.** | **P** | **Label** |
| --- | --- | --- | --- | --- | --- | --- | --- |
| Social support | <--- | Lockdown | .125 | .027 | 4.552 | *** | par_3 |
| Maladaptive cognition | <--- | Lockdown | .120 | .011 | 11.099 | *** | par_4 |
| Social support | <--- | Quarantine | -.272 | .070 | -3.916 | *** | par_5 |
| Maladaptive cognition | <--- | Quarantine | .073 | .027 | 2.691 | .007 | par_6 |
| MHP | <--- | Social support | -.067 | .003 | -25.818 | *** | par_8 |
| MHP | <--- | Maladaptive cognition | .287 | .007 | 42.896 | *** | par_9 |
| MHP | <--- | Lockdown | .007 | .007 | .967 | .334 | par_10 |
| MHP | <--- | Quarantine | .094 | .018 | 5.295 | *** | par_11 |
| General social support | <--- | Social support | 1.000 |  |  |  |  |
| Social support change | <--- | Social support | .525 | .017 | 31.087 | *** | par_1 |
| Rumination | <--- | Maladaptive cognition | 1.000 |  |  |  |  |
| Catastrophizing | <--- | Maladaptive cognition | 1.441 | .028 | 51.935 | *** | par_2 |
| Depression | <--- | MHP | 1.000 |  |  |  |  |
| Anxiety | <--- | MHP | 1.144 | .015 | 75.281 | *** | par_7 |

**Standardized Regression Weights: (Group number 1 - Default model)**

|  |  |  | **Estimate** |
| --- | --- | --- | --- |
| Social support | <--- | Lockdown | .037 |
| Maladaptive cognition | <--- | Lockdown | .089 |
| Social support | <--- | Quarantine | -.032 |
| Maladaptive cognition | <--- | Quarantine | .021 |
| MHP | <--- | Social support | -.230 |
| MHP | <--- | Maladaptive cognition | .392 |
| MHP | <--- | Lockdown | .007 |
| MHP | <--- | Quarantine | .038 |
| General social support | <--- | Social support | .791 |
| Social support change | <--- | Social support | .815 |
| Rumination | <--- | Maladaptive cognition | .764 |
| Catastrophizing | <--- | Maladaptive cognition | .861 |
| Depression | <--- | MHP | .856 |
| Anxiety | <--- | MHP | .907 |

**Variances: (Group number 1 - Default model)**

|  |  |  | **Estimate** | **S.E.** | **C.R.** | **P** | **Label** |
| --- | --- | --- | --- | --- | --- | --- | --- |
| **Lockdown** |  |  | .234 | .002 | 98.778 | *** | par_12 |
| **Quarantine** |  |  | .036 | .000 | 98.778 | *** | par_13 |
| **e7** |  |  | 2.680 | .094 | 28.642 | *** | par_14 |
| **e8** |  |  | .423 | .010 | 41.648 | *** | par_15 |
| **e9** |  |  | .181 | .003 | 54.559 | *** | par_16 |
| **e1** |  |  | 1.611 | .086 | 18.683 | *** | par_17 |
| **e2** |  |  | .373 | .024 | 15.810 | *** | par_18 |
| **e3** |  |  | .304 | .008 | 36.518 | *** | par_19 |
| **e4** |  |  | .310 | .016 | 18.912 | *** | par_20 |
| **e5** |  |  | .083 | .003 | 28.483 | *** | par_21 |
| **e6** |  |  | .064 | .004 | 17.253 | *** | par_22 |
