# Supplementary figures and images for: Social, Cognitive, and eHealth Mechanisms of COVID-19–Related Lockdown and Mandatory Quarantine That Potentially Affect the Mental Health of Pregnant Women in China: Cross-Sectional Survey Study
Source: J Med Internet Res. 2021 Jan 22;23(1):e24495. doi: 10.2196/24495 (PMC7836909; doi:10.2196/24495)

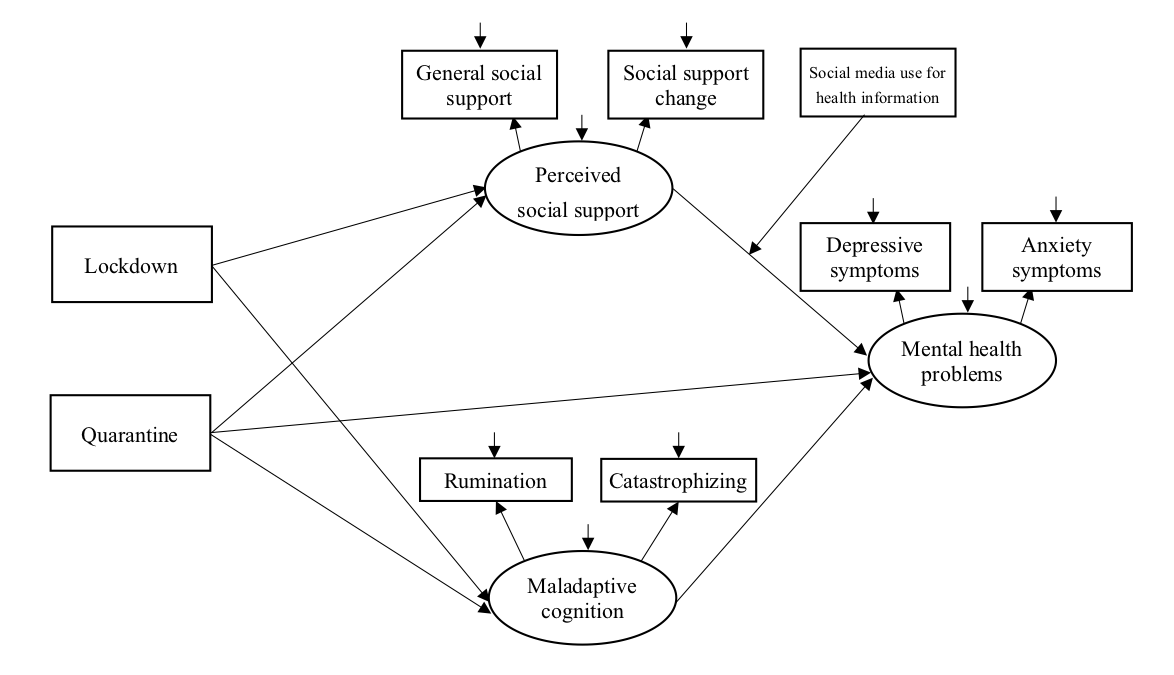

Supplement: Multimedia Appendix 2 [file jmir_v23i1e24495_app2.png]

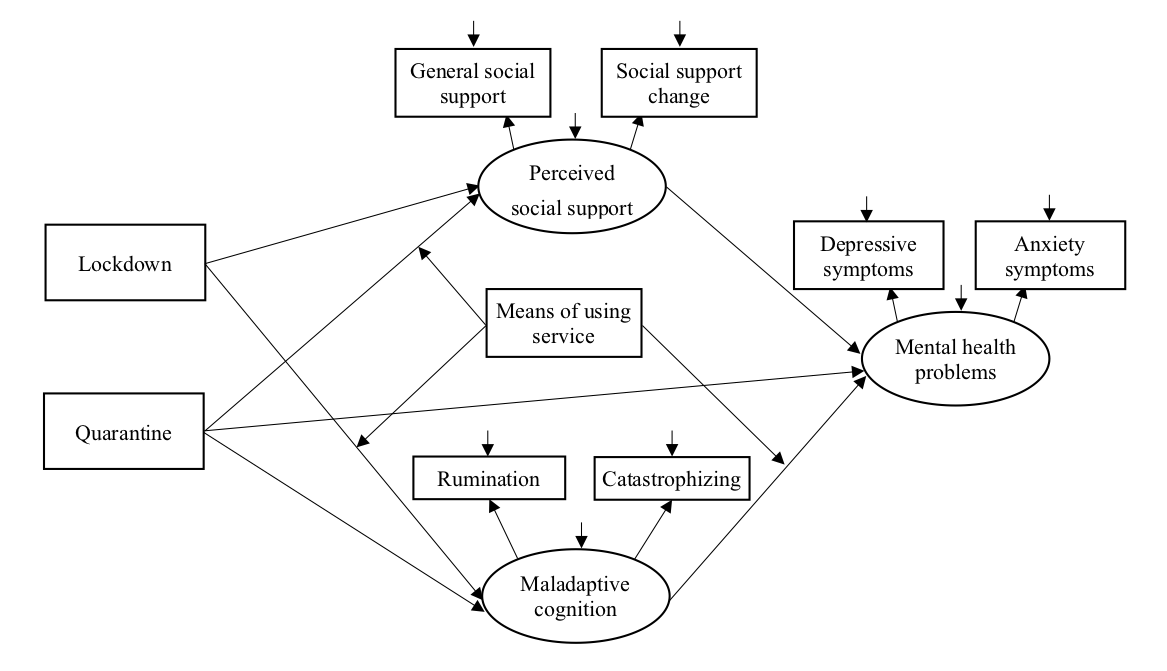

Supplement: Multimedia Appendix 3 [file jmir_v23i1e24495_app3.png]
